# Case Report: Tricuspid Annuloplasty for Right-Sided Congestive Heart Failure Secondary to Pulmonary Hypertension in a Dog

**DOI:** 10.3389/fvets.2022.843792

**Published:** 2022-02-22

**Authors:** Takeshi Mizuno, Kenta Sasaki, Sayaka Suzuki, Itsuma Nagao, Noriko Isayama

**Affiliations:** ^1^Veterinary Medical Center, Graduate School of Agricultural and Life Sciences, The University of Tokyo, Tokyo, Japan; ^2^Ve. C. Jiyugaoka Animal Medical Center, Tokyo, Japan; ^3^Uenonomori Animal Hospital, Tokyo, Japan; ^4^Japan Small Animal Medical Center, Saitama, Japan

**Keywords:** De Vega annuloplasty, cardiopulmonary bypass, tricuspid regurgitation, ascites, dog

## Abstract

An 11-year-old, 12.3-kg, female Miniature Dachshund was presented to our institution with ascites of unknown etiology. The dog had been administered moxidectin for 3 years to treat a heartworm infection. Thoracic radiographs showed enlargement of the right heart. Echocardiography revealed right atrial and ventricular dilatation as well as flattening of the interventricular septum. Heartworm was identified in the main pulmonary artery, which was dilated. Tricuspid regurgitation (TR) was observed using color Doppler ultrasonography, and 2.5 L of ascites were removed. The dog was diagnosed with pulmonary hypertension, severe TR, and right-sided congestive heart failure. Except at the initial site, heartworm was not detected using echocardiography, and the antigen test was negative. However, pharmacological treatment did not improve the right-sided congestive heart failure. Instead, De Vega tricuspid annuloplasty (TAP) was performed on the beating heart under cardiopulmonary bypass with the owner's consent. Sutures terminated between the two commissures in the middle of the annulus and were secured using another pledget. Annular reduction was performed by tying down the plication suture while the cylindrical sizer was inserted into the tricuspid valve orifice. The size of the cylindrical sizer was 16 mm, which was set based on the height and width of the septal leaflet. A 6-month follow-up showed a reduction of TR and right-sided volume overload with no evidence of ascites retention/recurrence or any other complication. Our findings indicate that TAP may be a valid treatment option for dogs with right-sided congestive heart failure caused by secondary TR.

## Introduction

Pulmonary hypertension (PH) leads to pressure overload in the right ventricle (RV), which induces right ventricular concentric and eccentric hypertrophy (1–4). Right ventricular eccentric hypertrophy can cause tricuspid regurgitation (TR) *via* annulus dilatation (1–4). Consequently, right-sided congestive heart failure (R-CHF) may develop (1, 2). The efficacy of therapy for dogs with PH is limited by the occurrence of right ventricular dysfunction, which results in a tricuspid annular plane systolic excursion (TAPSE) of <3.23 mm/kg^0.284^ or R-CHF (2). In human medicine, isolated TR secondary to PH is indicated for surgical treatment if the patient has symptoms of R-CHF (5). However, there are no guidelines for the treatment of TR in veterinary medicine. Furthermore, there are no reports of surgical treatment for acquired TR in dogs, although there are reports of surgical treatment for tricuspid dysplasia (6–8). To the best of our knowledge, this is the first report of a successful surgical tricuspid annuloplasty (TAP) performed in a dog with TR acquired secondarily to PH. In this report, we describe the successful treatment of TR secondary to PH using De Vega annuloplasty under cardiopulmonary bypass (CPB) in a dog.

## Case Presentation

An 11-year-old, 12.3-kg female Miniature Dachshund was presented to our institution to investigate and treat ascites of unknown etiology. The dog was diagnosed with filariasis at a primary hospital 3 years before presentation at our facility, for which moxidectin was continuously administered in the intervening period. Cardiac disease was thought to be the cause of the ascites, for which benazepril (0.2 mg/kg, BID) and pimobendan (0.12 mg/kg, BID) were prescribed at the primary hospital 4 months prior to presentation. The owners reported that the general condition of the dog was good. During the initial physical examination, the dog showed marked abdominal distension and a grade 2 systolic murmur over the right cardiac apex was noted.

### Diagnostic Assessment, Therapeutic Interventions, and Outcomes

**Figure 1:**
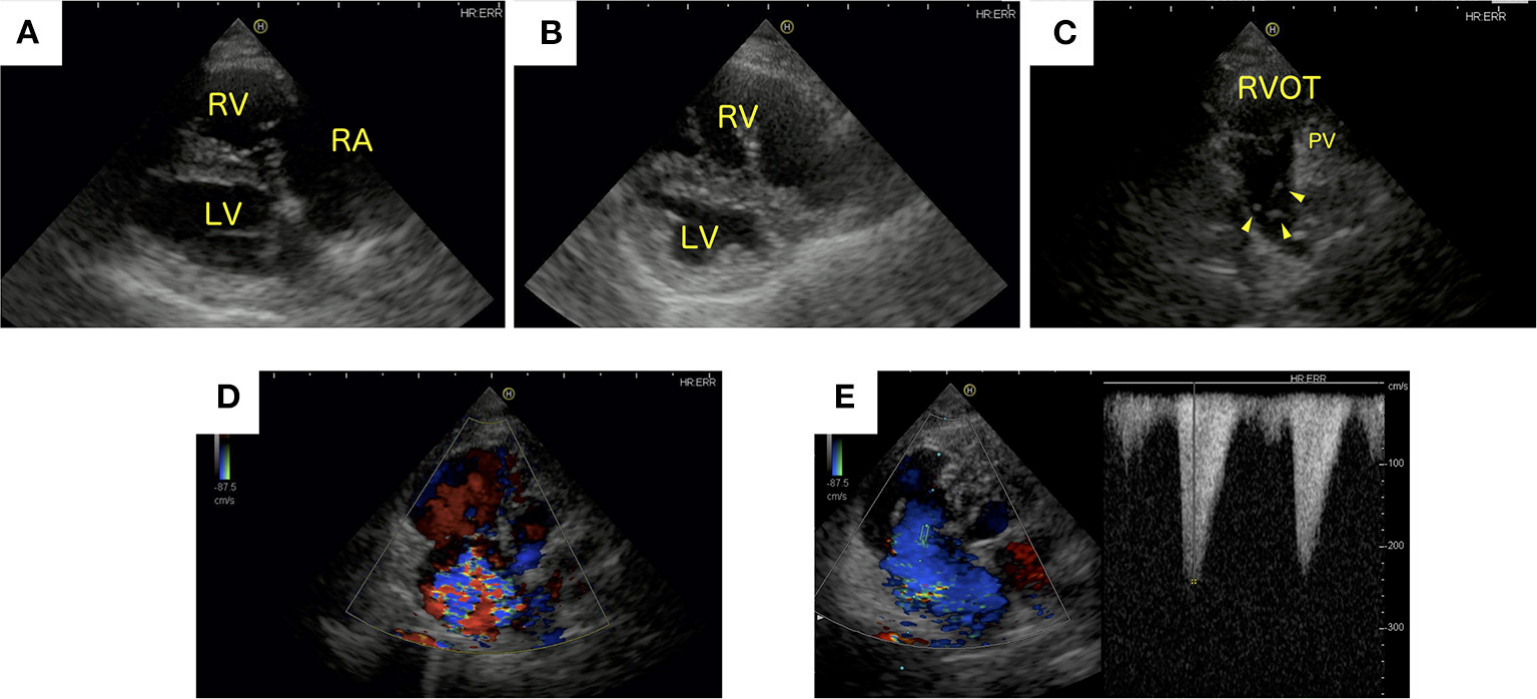
Echocardiographic images generated at initial presentation. **(A)** Right parasternal long-axis and **(B)** short-axis papillary muscle levels of the heart. **(C)** Left parasternal short-axis view of the heart. **(D)** Color Doppler image showing tricuspid regurgitation (TR) from a left apical, four-chamber view of the heart. **(E)** Continuous wave Doppler signal of the TR. **(A,B)** Right atrial and right ventricular dilatation and flattening of the interventricular septum was observed. **(C)** Heartworms were identified in the main pulmonary artery (arrowhead). **(D)** Severe TR was present. **(E)** Continuous wave Doppler examination of the tricuspid valve showing the characteristic dagger-shaped regurgitant signal. LV, left ventricle; PV, pulmonic valve; RA, right atrium; RV, right ventricle; RVOT, right ventricular outflow tract.

Radiographic evaluation of the thorax indicated right heart enlargement. Two-dimensional echocardiography showed right atrial and ventricular dilatation (Figure 1A). The septal leaflet of the tricuspid valve appeared slightly prolapsed. Flattening of the interventricular septum was also observed (Figure 1B). The main pulmonary artery was dilated (main pulmonary artery/aortic diameter = 1.42, reference range <1.0) (9). Echoes coming from filarial worms in the main pulmonary artery were identified (Figure 1C). Enlargement of the tricuspid valve annulus and a concomitant loss of coaptation were noted (Figure 1A). Continuous wave Doppler examination of the tricuspid valve returned a dagger-shaped regurgitant signal characteristic of severe TR (Figures 1D,E). The signal indicates a sharp decrease in the pressure gradient between the right atrium (RA) and RV and consequent elevation of right atrial pressure (10). The distensibility index of the right pulmonary artery was reduced (18.4%, reference range >35%) (11). The acceleration time (AT) was shortened, and the acceleration time/ejection time (AT/ET) ratio was reduced (AT = 50 ms, reference range 52–58 ms; AT/ET = 0.16, reference range >0.3) (1). The caudal vena cava collapse index was as low as 19% (12). A 2.5 L volume of ascites was removed. There was no evidence of respiratory disease or left-to-right shunting causing right ventricular enlargement. In addition, left-sided cardiac disease as a cause of post-capillary PH was not identified. Clinical findings and the clinical history supported a diagnosis of PH and secondary TR along with R-CHF.

Melarsomine is not available in Japan, and moxidectin had already been prescribed for 3 years leading up to initial presentation. Therefore, sildenafil and torsemide were prescribed as treatments for the PH and R-CHF. Despite the volume overload caused by TR and resultant right-sided congestive heart failure, the TAPSE value was low (2.8 mm/kg^0.284^) (2). Considering this, we concluded that the right ventricular systolic function was compromised, and we also prescribed pimobendan. Despite increasing the medication dosages [sildenafil (2.4 mg/kg per os, twice daily), pimobendan (0.25 mg/kg per os, twice daily), and torsemide (0.16 mg/kg per os, twice daily)], no improvement in R-CHF was observed. Removal of 2 L of ascites was required approximately every 2 weeks. On the 175th day of follow-up, the dog's activity and appetite decreased, and her general condition deteriorated. Echocardiographic data revealed persistent PH and right-sided volume overload. Except at the initial site, heartworm was not detected using echocardiography, and a heartworm antigen test was negative. In this case, the dog exhibited R-CHF rather than PH symptoms (e.g., syncope). Therefore, we determined volume overload caused by TR to be the main clinical concern. TAP under CPB was recommended. After obtaining the owner's consent, the surgical procedure was performed 221 days after initial presentation.

The anesthesia protocol used was the same as that described in a previous report (13). In preparation for CPB, the right carotid artery was surgically isolated. A right thoracotomy was performed at the 5th intercostal space after administering an intercostal nerve block with bupivacaine hydrochloride. The cranial and caudal vena cava were isolated and encircled with umbilical tape. A purse-string, 5-0 polypropylene suture was placed on the cranial and caudal aspect of the RA, respectively. The azygous vein was isolated and temporarily occluded with umbilical tape. Subsequently, heparin sodium (300 U/kg) was administered intravenously. After 5 min, the activated clotting time was measured and confirmed to be >300 s. An 8-Fr CPB cannula (Flexmate, Toyobo, Shiga, Japan) was inserted into the carotid artery to serve as the arterial line for the CPB. For venous return, a 12-Fr and 14-Fr CPB cannula (DLP Cardiopulmonary Bypass Cannula, Medtronic, Tokyo, Japan) was inserted into the cranial and caudal vena cava *via* the RA, respectively. CPB was achieved using a heart-lung machine (HAS-II, Senko Medical Instrument, Tokyo, Japan) with an extracorporeal circuit, venous reservoir, and membrane oxygenator (Exelung Kids, Senko Medical Instrument, Tokyo, Japan). After the air was removed from the CPB circuit and the activated clotting time was prolonged to 408 s, a partial CPB was initiated, and the dog's body temperature dropped to 28°C. Blood flow was set at 50–100 mL/kg/min on the CPB pump, and the cranial and caudal vena cava were occluded to initiate total CPB.

**Figure 2:**
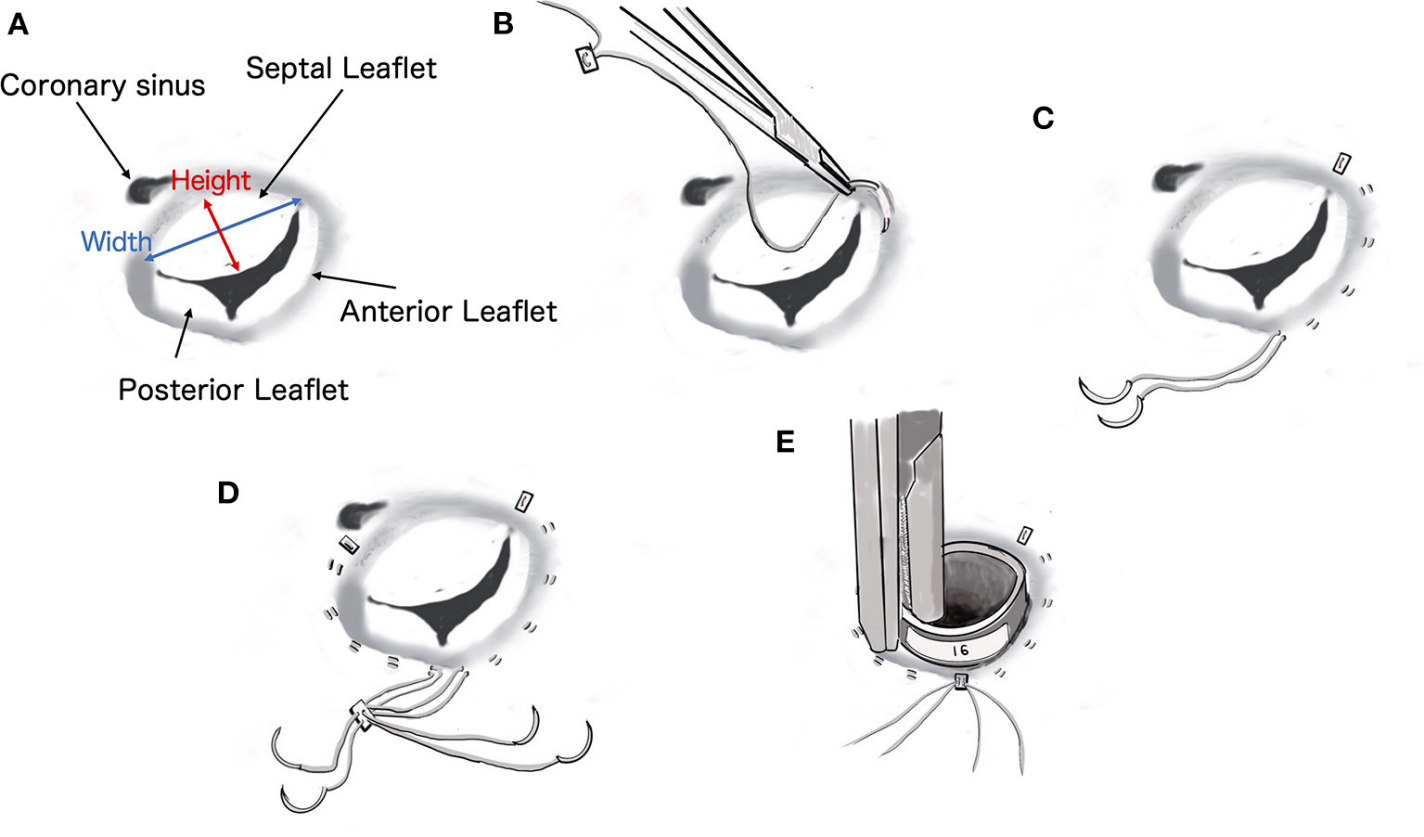
Diagrammatic representation of the procedure for De Vega tricuspid annuloplasty. The size of the cylindrical sizer was 16 mm, which was set based on the height and width of the septal leaflet (17 and 14 mm, respectively).

**Figure 3:**
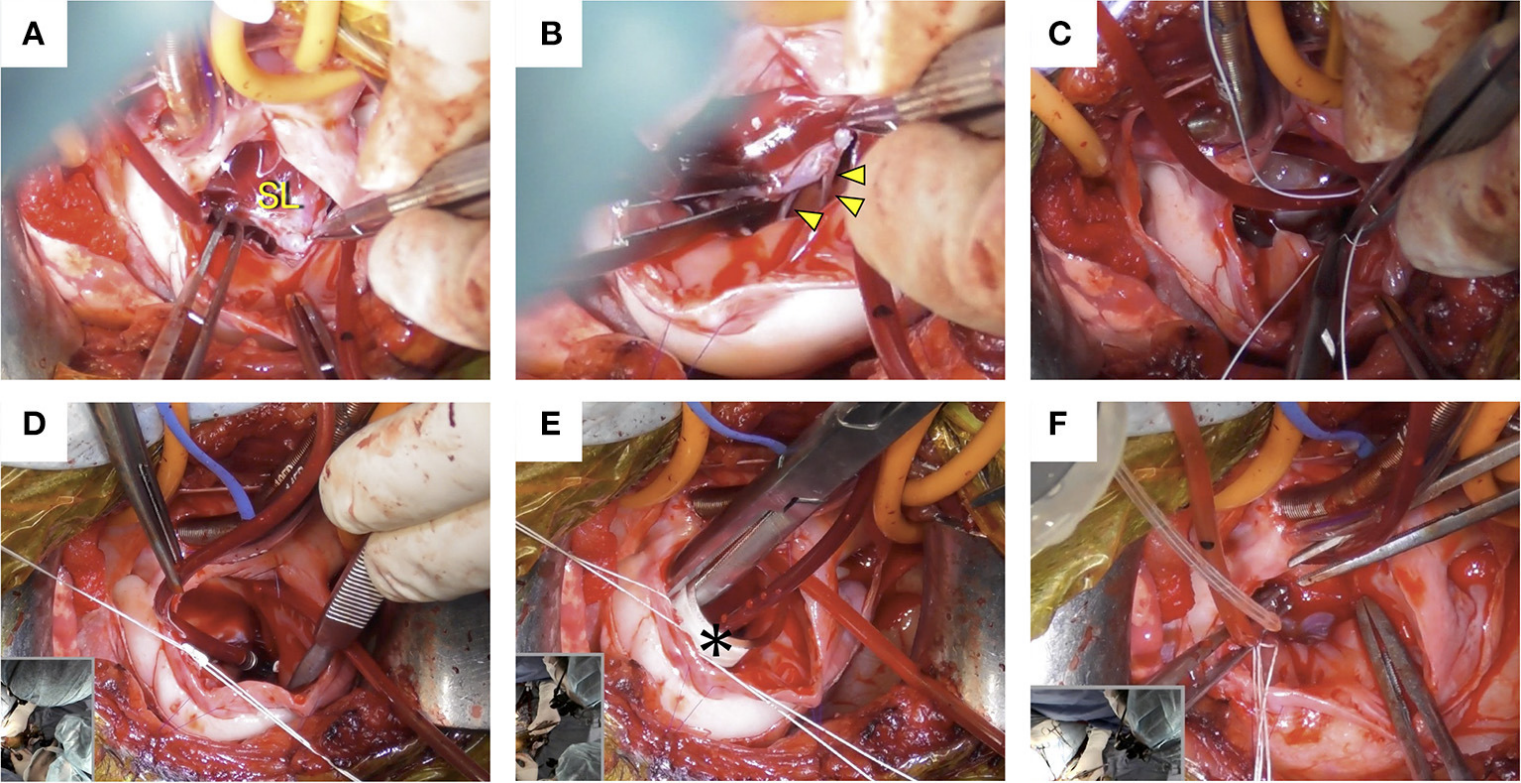
Intraoperative images. **(A)** The tricuspid valve was confirmed through the opened right atrium. **(B)** There was no elongation of the chordae tendineae in the tricuspid valve septum (arrowhead) or anterior and posterior leaflets, and no mobility problems were observed. **(C)** De Vega annuloplasty was performed with a CV-5 extended polytetrafluoroethylene suture. **(D)** Annular reduction was performed by tying down the plication suture while the cylindrical sizer was inserted into the tricuspid valve orifice. **(E)** The size of the cylindrical sizer (*) was 16 mm, which was set based on the height and width of the septal leaflet. **(F)** A regurgitation test after tricuspid annuloplasty was performed using saline, and the tricuspid valve was confirmed to be well closed. SL, septal leaflet.

Using a right atriotomy approach, intracardiac procedures were performed on the beating heart without administering cardioplegic solution. An incision was made from the RA to the right atrial appendage. The tricuspid valve was viewed through the opened RA (Figures 2A, 3A), and two filarial worms were removed from the RV. There was no elongation of the chordae tendineae in the septum of the tricuspid valve or anterior and posterior leaflets, and no mobility problems were observed (Figures 3A,B). Chordal reconstruction was deemed unnecessary, and only TAP using the De Vega technique was performed.

The De Vega annuloplasty was performed using CV-5 extended polytetrafluoroethylene (e-PTFE) sutures (GORE-TEX Suture, W.L. Gore & Associates, Newark, NJ). One e-PTFE suture with an e-PTFE pledget was started at the anteroseptal commissure and placed around the anterior aspect of the valve (Figures 2B,C). Another identical suture was started over the septal annulus, ~5 mm beyond the posteroseptal commissure, and placed around the lateral aspect of the valve (Figure 2D). Both sutures terminated between the two commissures in the middle of the annulus and were secured using another ePTFE pledget (Figure 2E). Annular reduction was performed by tying down the plication suture while the cylindrical sizer was inserted into the tricuspid valve orifice (Figures 3C,D). The size of the cylindrical sizer was 16 mm, which was set based on the height and width of the septal leaflet (17 and 14 mm, respectively; Figures 2A,E, 3E). A regurgitation test after TAP was performed using saline, and closure of the tricuspid valve was confirmed (Figure 3F). The RA incision was closed using continuous sutures of 6-0 polypropylene (Oval M, Matsudaika Kogyo, Tokyo, Japan). The dog's body temperature was brought back to 37°C, and CPB flow was gradually reduced before being completely turned off. Once the CPB was discontinued and the blood pressure was stable, the catheters were removed from the cranial and caudal vena cava and right carotid artery. A chest tube was inserted through the chest wall, and the cervical and thoracic incisions were closed using standard methods. To antagonize the effects of heparin, 4.5 mg/kg of protamine sulfate was administered intravenously over 30 min. Adequate blood pressure, oxygenation, and ventilation was then confirmed, and the anesthesia was reversed. The mean pre-operative venous pressure of 18 mmHg was decreased to 7 mmHg post-operatively. The durations of extracorporeal circulation, operation, and anesthesia were 70, 187, and 292 min, respectively. The dog recovered well with no anesthesia-related complications, and post-operative echocardiography revealed a reduction in tricuspid regurgitation. The dog was discharged on the eighth post-operative day and prescribed clopidogrel (2 mg/kg per os, once daily for 3 months), rivaroxaban (0.8 mg/kg per os, once daily for 1 week), sildenafil (2.1 mg/kg per os, twice daily), and pimobendan (0.2 mg/kg per os, twice daily).

Follow-up evaluations using echocardiography and chest radiography were performed at 1, 3, and 6 months after surgery.

**Figure 4:**
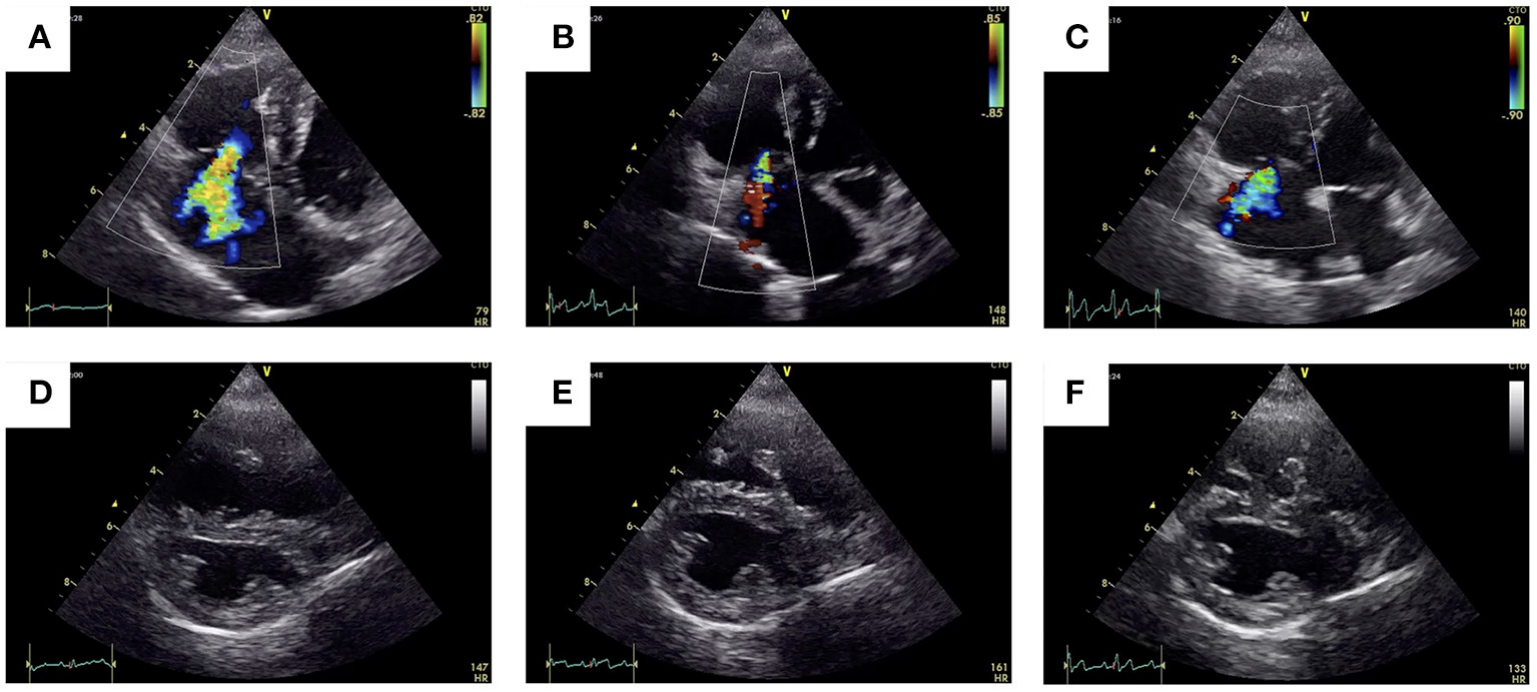
Pre-operative vs. post-operative transthoracic echocardiographic images. The 2-D color Doppler image of the tricuspid regurgitant jet obtained through the left apical imaging window **(A–C)** and of the flattening of the interventricular septum obtained using right parasternal short-axis views **(D–F)**.

At the 1-month follow-up, no ascites was observed, and the dog's condition was stable. Echocardiography showed good coaptation of the tricuspid valve and less interventricular septal flattening than was observed during the pre-operative period (Figures 4D,E). The eccentricity index in diastole was decreased (1.34) compared with the measurement taken during the pre-operative period (2.19). The right ventricular end-diastolic volume was reduced from 11.6 cm^2^ before surgery to 7.3 cm^2^. Color Doppler ultrasonography revealed minor tricuspid regurgitation, which was a notable improvement when compared with the pre-operative findings (Figures 4A,B). Compared with the pre-operative recordings, the post-operative fractional area change (FAC) and TAPSE values had decreased from 51.3 to 33.0% and 5.80 to 2.56 mm, respectively. There were no findings suspicious for thrombus formation.

At the 6-month follow-up, the ascites had been completely resolved, and the owner reported that the dog's general condition was good. Echocardiography revealed mild tricuspid regurgitation (Figures 4C,F), but the sizes of the RA and RV had not changed from 1-month follow-up (right ventricular end-diastolic volume = 7.3 cm^2^). The FAC and TAPSE values were 32% and 2.36 mm, respectively. Although there were improvements in the echocardiographic indices for PH, the presence or absence of PH could not be accurately assessed at follow-up because of reduced RV contractility. Therefore, we continued the treatment for PH beyond the 6-month follow-up. The dose of sildenafil was reduced to 1 mg/kg per os, twice daily, and pimobendan was continued at the same dose (0.2 mg/kg orally twice a day).

## Discussion

In human medicine, there are reports and guidelines regarding TAP for patients with isolated TR secondary to PH (5, 14, 15). However, there are no guidelines for the treatment of TR in veterinary medicine, and there are no reports of surgical intervention for acquired TR. In the current case, tricuspid valve annulus dilatation with a concomitant loss of coaptation were noted, but the prolapse of the tricuspid valve was mild. Thus, we decided on a diagnosis of TR secondary to PH. Volume overload caused by TR was favored over persistent heartworm infection as the primary clinical concern for the following reasons: the infection was no longer detectable using echocardiography except for at the initial site and pre-operative antigen tests were negative. Right-sided congestive heart failure caused by TR became refractory to medical management. Therefore, surgery for TR was recommended, and TAP was performed. Accompanying the post-operative decrease in TR, venous pressure also decreased, the ascites was resolved, and the patient's general condition improved. In this case, TAP was clinically successful and resulted in good patient outcome. Considering this, TAP may be an effective treatment for dogs with R-CHF caused by secondary TR.

In human medicine, TAPSE <17 mm has been associated with poor prognosis in patients with secondary TR (14, 15). Human patients with right ventricular dysfunction are indicated for surgery following careful consideration. In dogs, right ventricular dysfunction should also be carefully considered before initiating surgical intervention. However, there is a paucity of supporting evidence in veterinary medicine compared with in human medicine. Although pre-operative FAC was within the reference range in this case, there was concern about impairment of right ventricular function given the state of volume overload. The post-operative FAC was below the reference value, and the TAPSE was also reduced compared with pre-operative measurements (16). No post-operative complications occurred in this case because the surgery was performed on a beating heart out of concern for the possible right-heart dysfunction. However, the degree of right-heart dysfunction should always be determined as a measure of tolerance for surgical intervention in canine TR cases.

In this case, a De Vega rather than band annuloplasty was selected because the De Vega approach shortens the operation time and obtains good outcomes (8). De Vega annuloplasty has been shown to be effective and durable in the treatment of mitral regurgitation in dogs and has been performed in many cases (17, 18). However, use of this method for tricuspid valve surgery in dogs has not yet been reported. In human medicine, De Vega annuloplasty is useful for correcting functional TR with low long-term recurrence rates (16, 19–22). In human patients, the target annulus diameter is decided using an index calculated according to body surface area. This is done to avoid annuloplasty-related tricuspid stenosis and TR recurrence (23). However, there is no such index for tricuspid annuloplasty in dogs. Considering the possibility that annuloplasty may reduce the post-operative mobility of the anterior and posterior leaflets, the target tricuspid annulus diameter was set based on the height and width of the septal leaflet in this case. As a result, TR was well controlled. Further study is needed to examine and establish the appropriate annulus diameter for De Vega annuloplasty in canine patients. Expanded polytetrafluoroethylene sutures were used with the that expectation suture breakdown and dehiscence (the so-called bow-string effect) could be prevented (24), although evidence to this effect is lacking. Nishida et al. reported that e-PTFE has excellent tissue compatibility and durability and can be effectively used for canine mitral valve repair (25). In addition, we have recently used e-PTFE for mitral valve annuloplasty with good outcome (data not shown). In the present case, use of e-PTFE controlled the TR for 6 months, without breakdown of the suture annuloplasty. Overall, De Vega annuloplasty was useful for the treatment of TR in our canine patient. In conclusion, TAP may be a valid treatment option for TR acquired secondarily to PH in dogs.

## Limitations

A limitation of this study is about the indication of TAP for dogs with heartworm. TAP may also be a viable treatment option for TR secondary to PH. However, Calvert et al. reported no correlation between the number of infiltrating heartworms and pulmonary vascular resistance, indicating that host–parasite interactions play significant roles in the severity of disease (26). We acknowledge the possibility that removing the two heartworms intraoperatively may have contributed to clinical improvement. In this case, we could not perform adulticide therapy because melarsomine is unavailable in Japan. Pharmacological treatment may be prioritized over surgical intervention in environments where melarsomine is available. In dogs with heartworm, it is reported that once the endarteritis has developed, the vascular changes are chronic and may not be reversible (27). The reduction of TR cannot improve pulmonic vascular changes. Therefore, considering the consequences of heartworm in the pulmonary vasculature, it should be considered whether this surgery is an adequate therapeutic option for dogs with TR associated to PH in dogs with heartworm. Our findings suggest that TAP for TR with heartworm should be carefully considered when only treatments other than melarsomine are available. In the future, a more accurate evaluation of the efficacy of TAP will be supported by the performance of TAP in cases of TR secondary to PH with different etiologies.

## Data Availability Statement

The original contributions presented in the study are included in the article/supplementary material, further inquiries can be directed to the corresponding author/s.

## Ethics Statement

Ethical review and approval were not required for the animal study because this is a case report. Written informed consent was obtained from the owners for the participation of their animals in this study.

## Author Contributions

TM contributed to the conception and design of the work, analyzing the data, and writing the manuscript. KS and NI assisted with surgery and were involved with the aftercare and data acquisition. SS performed the general anesthesia. IN assisted with the pre-operative care. All authors contributed to the writing, editing, critical review, and approved the final version of the manuscript.

## Conflict of Interest

The authors declare that the research was conducted in the absence of any commercial or financial relationships that could be construed as a potential conflict of interest.

## Publisher's Note

All claims expressed in this article are solely those of the authors and do not necessarily represent those of their affiliated organizations, or those of the publisher, the editors and the reviewers. Any product that may be evaluated in this article, or claim that may be made by its manufacturer, is not guaranteed or endorsed by the publisher.
